# Prognostic Factors Associated With Sleep Duration: Serum Pro-Oxidant/Antioxidant Balance and Superoxide Dismutase 1 as Oxidative Stress Markers and Anxiety/Depression

**DOI:** 10.3389/ijph.2023.1606014

**Published:** 2023-09-07

**Authors:** Susan Darroudi, Mostafa Eslamiyeh, Karrar Khaleel Jaber Al-Fayyadh, Mohammad Zamiri Bidary, Sina Danesteh, Aliakbar Hassanzadeh Gouji, Reza Assaran Darban, Habibollah Esmaily, Majid Ghayour-Mobarhan, Mohsen Moohebati, Gordon A. Ferns

**Affiliations:** ^1^ International UNESCO Center for Health-Related Basic Sciences and Human Nutrition, Mashhad University of Medical Sciences, Mashhad, Iran; ^2^ Student Research Committee, Faculty of Medicine, Mashhad University of Medical Sciences, Mashhad, Iran; ^3^ Department of Biochemistry and Biophysics, Faculty of Sciences, Mashhad Branch, Islamic Azad University, Mashhad, Iran; ^4^ Social Determinants of Health Research Center, Mashhad University of Medical Sciences, Mashhad, Iran; ^5^ Cardiovascular Research Center, School of Medicine, Mashhad University of Medical Sciences, Mashhad, Iran; ^6^ Division of Medical Education, Brighton and Sussex Medical School, Brighton, United Kingdom

**Keywords:** anxiety, depression, cross-sectional study, sleep, PAB

## Abstract

**Objectives:** Sleep is a conserved vital behavior in humans, and insufficient sleep is associated with several disorders. Recent studies have investigated the association of sleep duration, oxidative stress markers, anxiety, and depression. Therefore, we aim to assess the relationship between sleep duration, serum pro-oxidant/antioxidant balance (PAB) and superoxide dismutase 1 (SOD1) levels as markers of oxidative stress, anxiety, and depression.

**Methods:** Participants included in our cross-sectional analysis were recruited as part of the MASHAD study (*n* = 9,184). Nocturnal sleep duration was identified using a self-reported questionnaire, and serum pro-oxidant/antioxidant balance (PAB) and superoxide dismutase 1 (SOD1) levels were assessed using methods that have been previously reported.

**Results:** Serum PAB, depression, and anxiety scores were found significantly higher in subjects with very short sleep duration. In an adjusted model using MANOVA regression analysis, serum PAB was significantly higher in the subjects with a very short sleep duration (*p*: 0.016 in depression and *p*: 0.002 in anxiety).

**Conclusion:** The present cross-sectional study demonstrates a relationship between sleep duration, oxidative balance, and depression/anxiety, especially in anxiety subjects that might predict each other.

## Introduction

Sleep deprivation refers to a state in which an individual gets less sleep than required to feel alert and awake [1]. It has been reported that 82.6% of retired Iranian elders suffer from poor sleep quality [2]. Moreover, from 2003 to 2012, sleep insufficiency was consistently increasing in children subjected to the National Survey of Children’s Health [3]. In support of the physiological importance of sleep, many studies have indicated that sleep deprivation is associated with several conditions, including psychiatric disorders, cardiovascular diseases, inflammation, metabolic disorders, immune dysfunction, and even premature death [4–9]. Furthermore, it is the leading cause of car accidents in Iran, accounting for the death of 20,000–80,000 individuals in Iran annually [10, 11]. Another recently proposed hypothesis for the adverse effects of sleep deprivation is the disturbance of pro-oxidant/antioxidant balance (PAB) in favor of oxidants, which leads to oxidative stress [12]. One study suggested that the superoxide dismutase1 (SOD1) enzyme, which is the first defense line against superoxide free radicals and also the most extensively studied antioxidant enzyme, is disrupted in individuals with sleep deprivation, which leads to oxidative stress (OS) [13]. Epidemiological studies have also suggested an association between sleep deprivation and affective disorders such as depression and anxiety in children and adolescents [14, 15]. Another study has suggested that early life sleep deprivation was a predictive risk factor for later onset of anxiety and depression [16]. According to the World Health Organization report in 2015, more than 322 and 264 million people are struggling with depression and anxiety disorders worldwide, making up 4.4% and 3.6% of the global population [17]. A systematic review showed that prevalence of depression and anxiety increased to 279.6 and 301.4 million people [18]. Moreover, the incidence of these two common psychiatric disorders is increasing, especially in countries with low or middle income [19]. Depression is the single largest, and anxiety is the sixth most significant cause of disability and health loss globally [19].

To date, there has been no consensus on the physiological functions of sleep. A controversial hypothesis for the physiological role of sleep is acting as an antioxidant mechanism for the brain, which was proposed by Reimund [20]. The Reactive Oxygen Species (ROS) and oxidative stress markers could be accumulated in the brain tissue during Sleep deprivation [21]. Some stud suggested, enough sleep can increase antioxidant activity which promotes a brain protection against free radicals via a decrease in oxidant production [22]. Oxidative stress can also result in a wide range of diseases such as neurodegenerative diseases, cancer, male infertility, diabetes, autoimmune diseases, and cardiovascular disorders [4, 23–27]. In the past few years, numerous studies have sought to evaluate the involvement of OS in psychiatric disorders. Predominantly, therapeutic methods for depression and anxiety have focused on gamma-aminobutyric acidergic and serotoninergic systems [28]. But, researchers have established a link between OS and depression/anxiety during the last few years, suggesting that other systems like oxidative metabolism can affect mental health, which might draw more attention to antioxidants [28–30]. Nonetheless, there remains a controversy surrounding the association of depression/anxiety with PAB. It is not yet well understood whether depression/anxiety are associated with increased or decreased PAB [31].

**FIGURE 1:**
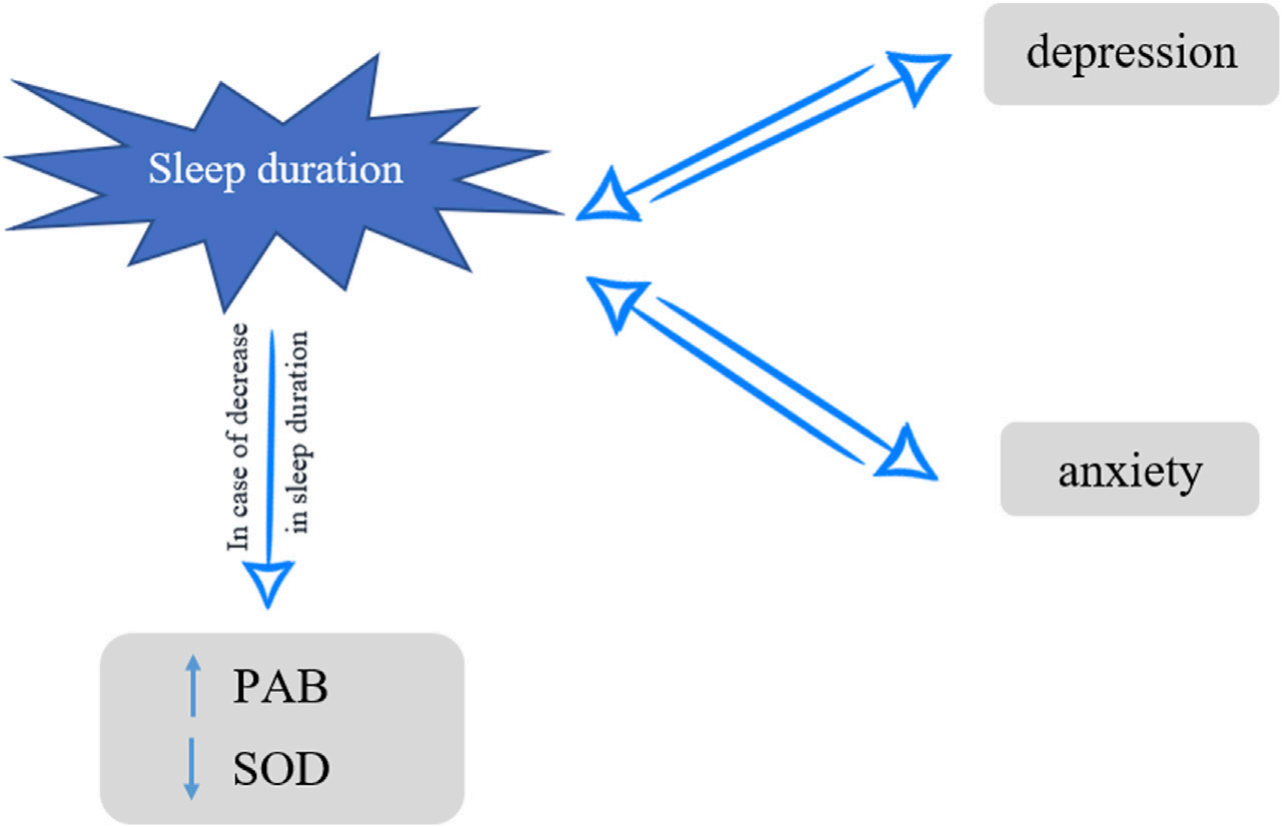
Nightly sleep, oxidative stress and depression/anxiety, Mashhad stroke and heart atherosclerotic disorders, Iran, 2010–2020.

Understanding the physiological functions of sleep and its role in antioxidant mechanisms is necessary for identifying the impacts of sleep deprivation on mental health. Since most studies regarding the effect of sleep deprivation on PAB and anxiety/depression and the association between serum PAB and anxiety/depression are mostly conducted on animals or non-population-based with limited sample sizes, we cannot have a decisive conclusion. Consequently, the primary objective of this population-based cross-sectional study was to investigate the impact of sleep duration on PAB, SOD1, anxiety, and depression. The secondary objective was to evaluate the association of these oxidative stress determinants with anxiety and depression in subjects with very short nightly sleep (Figure 1).

## Methods

### Study Population

This research study was conducted on subjects recruited as part of the Mashhad stroke and heart atherosclerotic disorders (MASHAD) 10 year cohort study, which was started in 2010 and is continued up to 2020 on 9,704 Mashhad citizens selected from three regions of Mashhad, a highly populated city in the northeast of Iran. MASHAD study characteristics and methodology was discussed in detail in the previously published manuscript [32]. The inclusion criteria was age 35–65 and no history of CVD. The exclusion criteria for recruitment at baseline including no history of CHD, cancer, chronic kidney disease.

### Ethics Approval and Consent to Participate

This study was approved by the Ethics Committee of the Mashhad University of Medical Sciences (MUMS) (MASHAD study code: 85134). All methods were performed in accordance with the relevant guidelines and regulations. Informed consent was obtained from all subjects in the ethics approval and consent to participate. The ethic approval was IR.MUMS.REC.1386.250.

### Sleep Measurement

We assessed the nocturnal sleep duration of our participants using a questionnaire (self-report). Thereafter, subjects were put in three defined subgroups: <5 (very short sleep), 5–7 h (short sleep), and ≥7 h (normal and long sleep). Night shift workers and subjects in the very short sleep (<5 h) subgroups who sleep in the afternoons were not included in this cross-sectional analysis [33].

### Serum PAB Measurement

Measuring all oxidants and antioxidants separately is costly and time-consuming. The PAB assay, developed by Hamidi et al., is a technique that simultaneously measures oxidants and antioxidants in a single test [34]. A standard curve was provided by the values determined in the standard samples. The PAB values were expressed in arbitrary HK (Hamidi-Koliakos) unit, which are the percentage of hydrogen peroxide in the standard solution. Finally, the values of the unknown samples were calculated based on the values obtained from the standard curve.

### Serum SOD1 Assay

The activity of superoxide dismutase-1 activity was assessed by pyrogallol solution and Tris–cacodylic acid buffer under the conditions explained [35].

### Depression and Anxiety Assessment

For measuring depression in all participants, Beck Depression Inventory (BDI) [36] questionnaire has been applied in which consisted of 21 items, and each item was scored from 0 to 3. Based on the questionnaire results, all subjects were scored from 0 to 63, and scores in 0–13, 14–19, 20–28, and 29–63 were considered and categorized into minimal, mild, moderate, and severe depression subgroups, respectively [37]. Furthermore, for assessing anxiety symptoms, we used Beck Anxiety Inventory (BAI) [38]. This questionnaire was mainly used to evaluate anxiety symptoms frequency, but due to previous results, it was utilized to identify the severity of anxiety in this study subjects [39]. The same as BDI, the BAI questionnaire had 21 items with a score of 0–3 for each item, and totally 0 to 63 points were given to each subject. Participants with a score of 0–7, 8–15, 16–25, and 26–63 were divided into minimal, mild, moderate, and severe anxiety subgroups, respectively [38]. Previously, the Persian version of BDI and BAI questionnaires was validated appropriately by Ghassemzadeh et al. [40] and Kaviani and Mousavi [41] studies, respectively.

### Statistical Analysis

Data were analyzed using two-sided tests using SPSS version 23 (SPSS Inc. Released 2009. PASW Statistics for Windows, Version 23.0. Chicago: SPSS Inc.). Quantitative and qualitative variables are reported as mean ± SD and number (percentage), respectively. Kolmogorov-Smirnov analytical test was performed to check whether the data are normally distributed or not. Chi-square analysis was performed in order to assess differences between subgroups of two qualitative variables, and we used a one-way analysis of variance (ANOVA) test for the determination of differences in the amount of normally-distributed quantitative variables according to subgroups of nightly sleep duration or anxiety or depression severities. Moreover, we applied the multivariate analysis of variance (MANOVA) test to evaluate changes in the serum level of PAB and SOD1 in the subgroups of nightly sleep duration. We defined *p*-value under 0.05 as significant. Graph pad prism was used for drawing figures.

## Results

In the current analysis, 9,184 subjects were eligible and enrolled, of whom 3,682 (40.09%) were male. As indicated in Table 1, a chi-square analysis found men were significantly more likely to experience short sleep duration than women. In addition, one-way ANOVA indicated a significant difference in age, depression and anxiety scores, and serum PAB level between three defined subgroups of nightly sleep duration. After performing *post hoc* analysis, we found that anxiety and depression scores are meaningfully higher in very short sleep subjects in comparison with the other groups. Furthermore, for PAB, it was considerably higher in the very short sleep subgroup than just normal subjects. No significant differences were found in all mentioned features between short and normal participants.

**TABLE 1:** Study participants’ demographic characteristics and oxidative stress determinants based on their nightly sleep duration in Mashhad stroke and heart atherosclerotic disorders, Iran, 2010–2020.

	Very short sleep <5 h	Short sleep 5–7 h	Normal sleep >7 h	*p*-value
Number	*N* = 456, 4.96%	*N* = 2,968, 32.31%	*N* = 5,760, 62.71%	
Age, years	51.27 ± 7.76	48.99 ± 7.97	47.25 ± 7.96	**<0.001**
Sex, N (%)				
Male	176 (4.78)	1,248 (33.89)*	2,258 (61.32)	**0.029**
Female	280 (5.08)	1,720 (31.26)	3,502 (63.64)	
Depression score	16.67 ± 11.93	13.17 ± 9.85^a^	11.77 ± 9.26^a^	**<0.001**
Anxiety score	15.74 ± 12.84	11.31 ± 10.08^a^	9.85 ± 9.24^a^	**<0.001**
SOD1, IU	2.22 ± 1.86	2.15 ± 1.8	2.12 ± 1.78	0.46
PAB, HK	72.16 ± 58.51	68.76 ± 53.46	65.77 ± 55.23^a^	**0.001**

*significant differences is related to this group. a: <5 h vs. 5–7 h and >7 h. **Chi-square** test has been done for evaluating differences of sex distribution in subgroups of nightly sleep duration and **one-way ANOVA** was performed for analysis of the other variables; Mean and Standard Deviation (SD) of the quantitative data in the target population is represented as Mean ± SD; PAB, pro-oxidant Antioxidant Balance; SOD1, superoxide dismutase 1.

**FIGURE 2:**
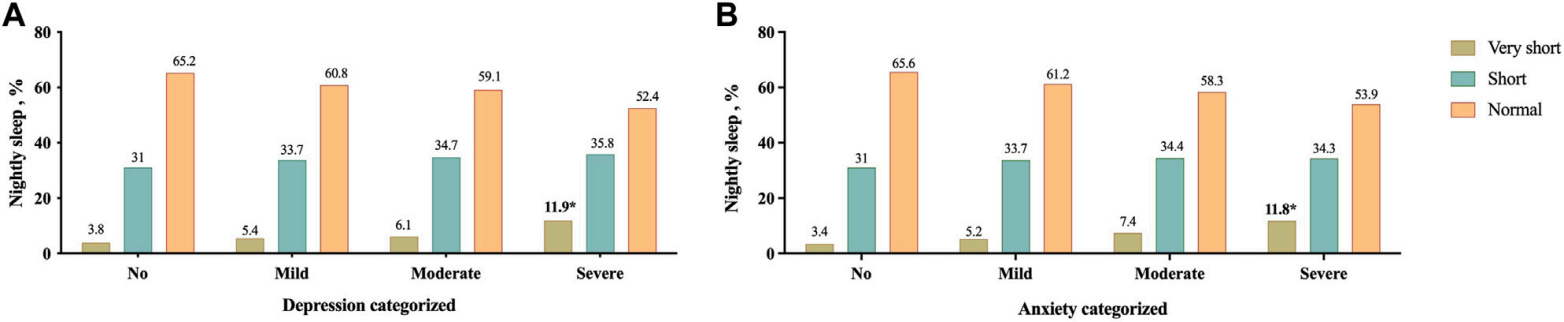
Prevalence of different severity categories of depression **(A)** and anxiety **(B)** based on their nightly sleep duration; *p < 0.05, Mashhad stroke and heart atherosclerotic disorders, Iran, 2010–2020.

**FIGURE 3:**
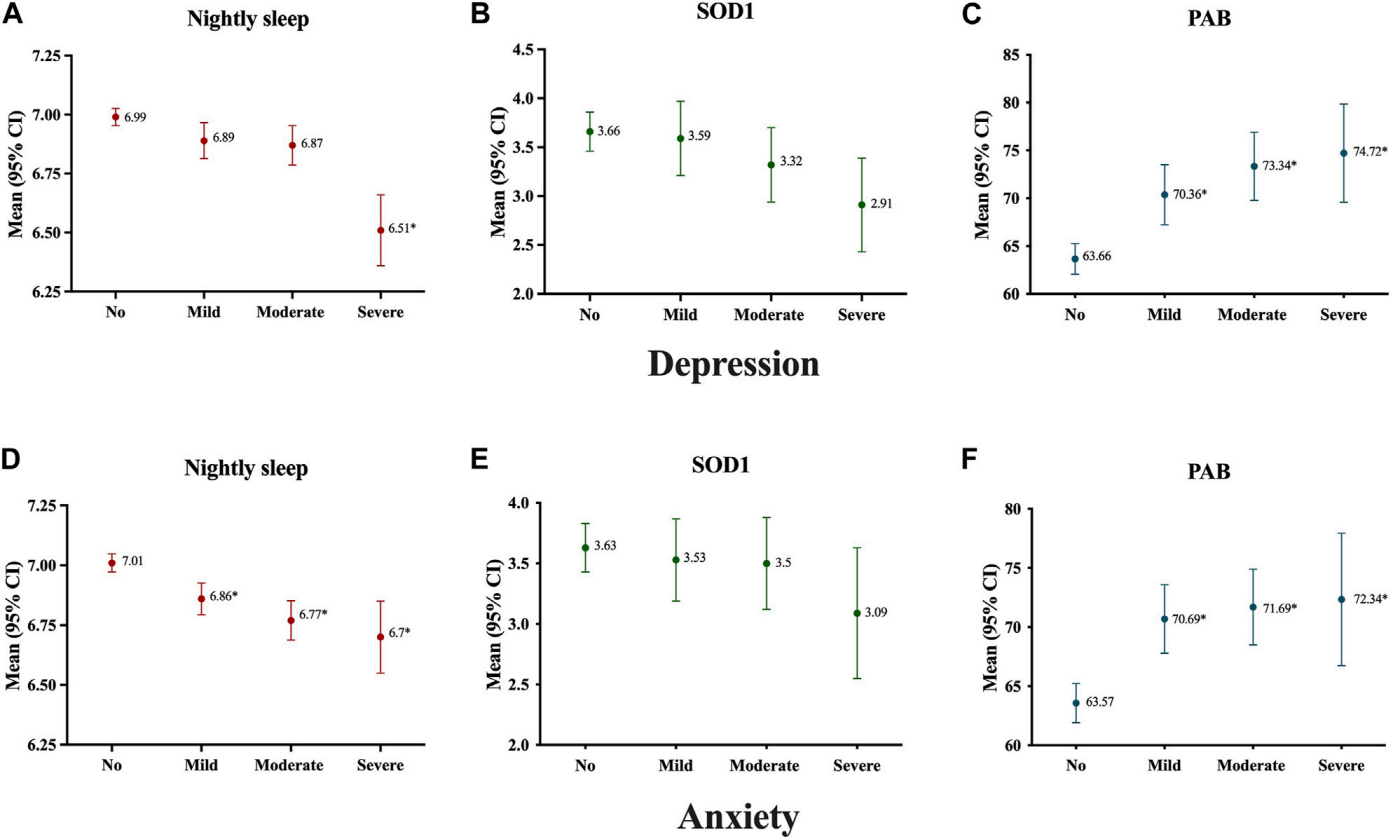
Antioxidant status and nightly sleep duration of study population according to severity of subjects’ depression **(A–C)** and anxiety **(D–F)**; Data presented as mean ± 2SE; *p < 0.05, Mashhad stroke and heart atherosclerotic disorders, Iran, 2010–2020.

Based on results showed in Figure 2, in participants with very short nightly sleep, severe cases were found significantly more frequent depression and anxiety than the other groups. Moreover, as shown in Figure 3, the nightly sleep duration and serum PAB level were respectively, significantly lower and higher in severe depressive and anxiety cases than the other ones.

**FIGURE 4:**
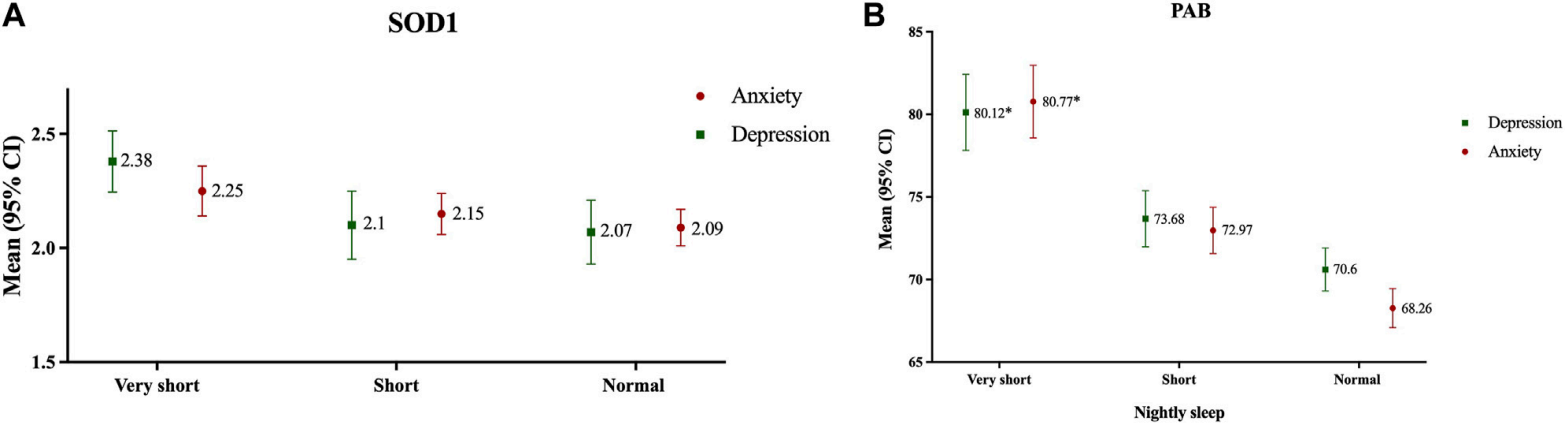
Antioxidant status according to nightly sleep duration in subjects with depression and anxiety [**(A)**: SOD; **(B)**: PAB]; Data presented as mean ± 2SE; *p < 0.05, Mashhad stroke and heart atherosclerotic disorders, Iran, 2010–2020.

In crude analysis, it was found that in both depressive and anxiety subjects, serum PAB was significantly higher in very short sleep subjects in comparison with the others, respectively (Figure 4). After adjusting results for age and sex in a MANOVA regression analysis, we found that subjects with anxiety and depression and very short sleep had 12.09- and 10.69-unit PAB more than others, respectively (Table 2).

**TABLE 2:** Impact of nightly sleep duration on serum level of PAB and SOD1 in subjects with depression and anxiety separately, respectively adjusted for age and sex in Mashhad stroke and heart atherosclerotic disorders, Iran, 2010–2020.

	Depression subjects			
	Nightly sleep duration	Beta coefficient	SE	*p*-value
Serum PAB	>7 h (Ref.)			
	5–7 h	3.822	2.264	0.092
	<5 h	10.696	4.456	0.016
Serum SOD1	>7 h (Ref.)			
	5–7 h	0.026	0.069	0.75
	<5 h	0.31	0.128	0.15
	**Anxiety subjects**			
Serum PAB	>7 h (Ref.)			
	5–7 h	3.921	1.93	**0.042**
	<5 h	12.092	3.882	**0.002**
Serum SOD1	>7 h (Ref.)			
	5–7 h	0.45	0.073	0.42
	<5 h	0.12	0.151	0.54

Multivariate Analysis of Variance (MANOVA) test was performed; SE, standard error.

## Discussion

We have demonstrated that very short nightly sleep was significantly associated with higher depression and anxiety scores. There was a significant association between sleep duration and serum PAB level, but serum SOD1 activity was not associated with sleep duration. In individuals with Generalized Anxiety Disorder (GAD), sleep duration was conversely associated with PAB. In other words, the less sleep duration, the higher PAB.

In 1994, Reimund suggested that a key function of sleep might be acting as an antioxidant for the brain [20]. Oxidative phosphorylation -taking place in mitochondria-is the main ATP supply of the cell in all aerobic organisms that produce free radicals as a by-product [31]. At low concentrations, free radicals are necessary for normal cell functioning. They serve numerous physiological processes, including mitosis, gametogenesis, cell signaling, and cellular response to infection and injury [25, 42]. When the disturbance in pro-oxidant/antioxidant balance (PAB) occurs in favor of the oxidants, cells may be exposed to oxidative stress. OS results from either increased generation of reactive oxygen species (ROS) or impaired oxidative defense mechanisms. SOD is the most extensively studied antioxidant enzyme and serves as the first defense line against superoxide free radicals, which catalyzes the dismutation of the highly reactive superoxide radical, O^2−^, to O_2_ and H_2_O_2_ [13, 31]. The brain is especially more susceptible to OS because it is mostly made up of lipid and consumes comparatively large amounts of oxygen and produces an excessive amount of free radicals with a relatively modest oxidative defense system [43]. In support of the Reimund hypothesis, Hill and associates have indicated that the upregulation of gene expression of antioxidant enzymes in wild-type flies shortens sleep duration, suggesting that decreasing PAB promotes wakefulness [44]. One previous study has reported that sleep deprivation may induce oxidative stress, suggesting that sleep clears the ROS accumulated in the brain during wakefulness [45].

We found that inadequate sleep is associated with a high serum PAB in subjects with anxiety and depression. To our knowledge, we are the first study to show this association. In contrast to our findings, in studies conducted on humans, OS was not observed in sleep-deprived individuals [46–48]. Furthermore, in a narrative review, Atrooz et al. have suggested that while chronic sleep deprivation diminishes the antioxidant response, acute sleep deprivation may augment antioxidant mechanisms [13]. There are inconsistent results regarding the impact of sleep deprivation on SOD1 activity. In line with our findings, Gopalakrishnan and coworkers found no significant change in SOD1 activity in rats with chronic sleep deprivation [46].

As previously discussed, the brain is especially vulnerable to oxidative stress (OS); and OS can affect synaptic integrity, neurotransmission, and the overall function of the brain, resulting in neurological and psychological disorders [49, 50]. Data on the relationship between OS and depression/anxiety are controversial. In line with our findings, some authors have revealed a strong positive relationship between OS and depression/anxiety [28, 51, 52]. Contrastingly, some authors have failed to demonstrate an association between OS and the presence of depression/anxiety [53–55]. When it comes to the activity of antioxidant enzymes in depression/anxiety, the data are discrepant. While we found no differences in serum SOD1 activity in different depression/anxiety severities, similarly, as observed in another study, both increased and decreased activity of SOD1 have been reported previously [31, 56, 57]. An increased expression of SOD1 may induce inflammation, which inflammation itself can contribute to depression/anxiety [29, 58, 59]. Far as we know, researches evaluating the association between different depression/anxiety severities and OS using PAB are scarce. In contrast with our results, in another study, no association was found between serum PAB level and severity of depression [55].

Our study has assessed the trinary relationship between subjects’ sleep duration, oxidative stress, and anxiety/depression scores of a large randomly-selected population in a city in the Middle East. In addition to our considerable efforts, some limitations existed in our procedures. First, our analysis was a cross-sectional one that could not accurately conclude cause and effect relationships between these three mentioned features. Moreover, data on the nocturnal sleep duration of our participants were collected using self-report questionnaires and we could not assess other oxidative stress parameters. Further cohort and case-controlled studies with more accurate methods for observation of sleep are required to better identify the risks of sleep deprivation.

In conclusion, our cross-sectional analysis revealed a logical trinary association between sleep duration, oxidative stress, and anxiety/depression and mainly indicated that sleep deprivation is associated with increased pro-oxidant/antioxidant balance and severe depression/anxiety. For future works we can use another categorization for response variables and also we can use machine learning algorithms for analyzing the data.

## Data Availability

The datasets used and/or analyzed during the current study available from the corresponding author on reasonable request.
